# Identification of ASCL1 as a determinant for human iPSC-derived dopaminergic neurons

**DOI:** 10.1038/s41598-021-01366-4

**Published:** 2021-11-15

**Authors:** Aaron M. Earley, Lena F. Burbulla, Dimitri Krainc, Rajeshwar Awatramani

**Affiliations:** 1 Ken & Ruth Davee Department of Neurology, Northwestern University Feinberg School of Medicine, Chicago, IL 60611 USA; 2 German Center for Neurodegenerative Diseases (DZNE), Munich, Germany; 3 Metabolic Biochemistry, Biomedical Center (BMC), Faculty of Medicine, Ludwig-Maximilians University, Munich, Germany; 4 Munich Cluster for Systems Neurology (SyNergy), Munich, Germany

**Keywords:** Induced pluripotent stem cells, Developmental neurogenesis, Neuronal development, Stem-cell differentiation, Stem-cell differentiation

## Abstract

During cellular specification, transcription factors orchestrate cellular decisions through gene regulation. By hijacking these transcriptional networks, human pluripotent stem cells (hPSCs) can be specialized into neurons with different molecular identities for the purposes of regenerative medicine and disease modeling. However, molecular fine tuning cell types to match their in vivo counterparts remains a challenge. Directing cell fates often result in blended or incomplete neuron identities. A better understanding of hPSC to neuron gene regulation is needed. Here, we used single cell RNA sequencing to resolve some of these graded molecular identities during human neurogenesis from hPSCs. Differentiation platforms were established to model neural induction from stem cells, and we characterized these differentiated cell types by 10x single cell RNA sequencing. Using single cell trajectory and co-expression analyses, we identified a co-regulated transcription factor module expressing achaete-scute family basic helix-loop-helix transcription factor 1 (ASCL1) and neuronal differentiation 1 (NEUROD1). We then tested the function of these transcription factors in neuron subtype differentiation by gene knockout in a novel human system that reports the expression of tyrosine hydroxylase (TH), the rate limiting enzyme in dopamine synthesis. ASCL1 was identified as a necessary transcription factor for regulating dopaminergic neurotransmitter selection.

## Introduction

Human induced pluripotent stem cells (iPSCs) and embryonic stem cells (ESCs) provide unique access into studying developmental mechanisms of cellular specification. Realization of this goal requires establishing directed differentiation model systems with high fidelity in recapitulating natural processes of the human embryo. There have been significant advances in establishing defined culture conditions for neural specification from iPSCs and ESCs^1–4^. Many neural directed differentiations inhibit bone morphogenic protein (BMP) and nodal-activin-transforming growth factor beta (TGFβ) pathways with the small molecules LDN-193189 and SB-431542, also known as dual-SMAD inhibition^1^. The dual-SMAD inhibition rapidly converts pluripotent stem cells to adopt neural progenitor identities within a week of culture^1^.

Modifications to the dual-SMAD culture platform have been claimed to bias neural progenitors to acquire type specific neurotransmitter identities and regionalized molecular signatures^1,5–14^. Despite these studies, the molecular regulation of neurotransmitter selection in iPSC/ESC-derived neurons is not well understood. Previous protocol optimization to direct specific neurotransmitter phenotypes has been a highly iterative process and reliant on population transcriptomics, which lacks information about the cellular diversity of a given system. To gain further insights into how neural precursors adopt unique neurotransmitter identities, we used single cell RNA profiling to resolve heterogeneity of neural cultures programmed from human iPSCs. We then used these molecular profiles to identify transcriptional regulators of specific neurotransmitter type neurons. In this study, we address regulation of the iPSC-derived dopaminergic phenotype.

Recent single cell RNA sequencing of the mammalian nervous system suggests that genes related to neurotransmission are main “drivers” of neuronal diversity^15^. Different nervous system regions present unique neurotransmitter codes and levels of neurotransmitter co-expression. Human and mouse midbrain dopaminergic neurons, which originate from the ventral midbrain^16–20^, have been defined by single cell RNA sequencing^21–24^. This single cell data has provided some insights into the expression of transcription factors that may regulate neuronal lineages. Early post-mitotic cells within the dopaminergic lineage in mouse and human express basic helix-loop-helix (bHLH) and helix-loop-helix (HLH) transcription factors such as NEUROD1, NEUROD2, ASCL1, neurogenin 2 (NEUROG2), and nescient helix-loop-helix 1 (NHLH1)^21^. Some of these transcription factors have been designated as “pioneer transcription factors” that can access and regulate chromatin landscapes during neural specification^3,4,25–29^. Loss of function studies of the bHLH transcription factors NEUROG2 and ASCL1 in mouse have suggested specificity of bHLH function in dopaminergic development^30,31^. It is also known that regionally distinct mouse dopaminergic neurons depend on different bHLH transcription factors for proper differentiation^30,32^. However, little is known about how bHLH transcription factors regulate neurogenesis and neurotransmitter identities in human iPSC-based differentiations.

In this paper, we analyze single cell expression of dual-SMAD based directed differentiations in a human iPSC model system. We harnessed the intrinsic cellular heterogeneity of this system to predict cellular lineages with single cell trajectory analysis. Candidate bHLH transcription factors that regulate neuron commitment were identified and prioritized for dopaminergic types based on a fluorescently activated cell sorting (FACS)-single cell RNA sequencing method. Finally, we performed loss of function experiments on prioritized transcription factors, ASCL1 and NEUROD1, to study their effect on the iPSC-derived dopaminergic phenotype. Our findings suggest ASCL1 is necessary for efficient dopaminergic neurotransmitter acquisition during iPSC to neuron cellular programming.

## Results

During nervous system development, several signaling cascades regulate cellular specification including BMP, wingless-related integration site (Wnt), fibroblast growth factor (FGF), sonic hedgehog (Shh), and retinoic acid (RA) pathways. These pathways pattern neuroepithelial cells into neural progenitor cells, known as radial glia, and through symmetric and asymmetric cellular divisions, specialize into diverse neural cell types^33,34^. We asked whether we could recapitulate neuronal differentiation from radial glial cells in a human iPSC model system. To address this, we established a human iPSC-derived radial glial/ neuronal mixed culture model system and characterized these cultures using 10x single cell RNA sequencing.

**Figure 1:**
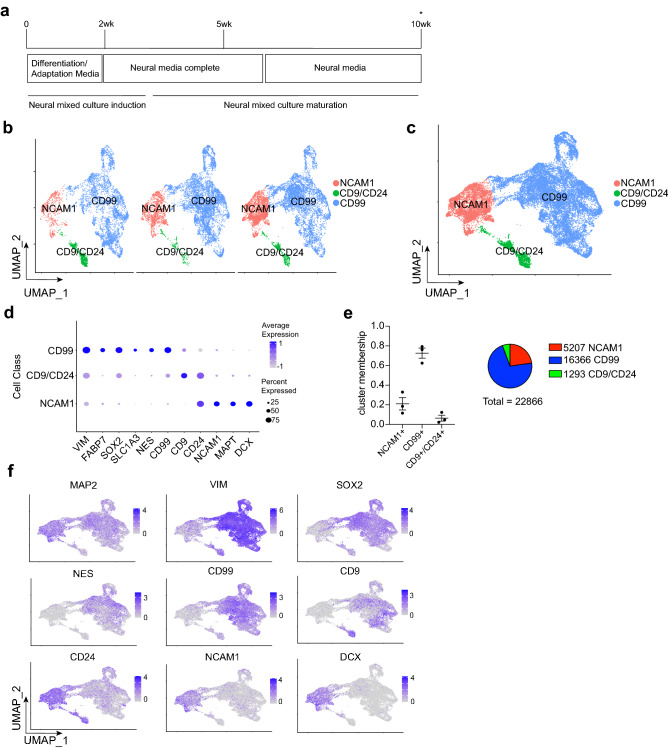
10x single cell RNA sequencing of a human iPSC-derived neural mixed culture. (**a**) Neural mixed culture differentiation timeline (complete method of chemical induction described in “Materials and methods” section). *Time of harvest for 10x single cell RNA sequencing. (**b**) UMAP of integrated cell type clustering (*n* = 3 independent differentiations) with cell classes defined by cell surface markers. (**c**) Merged UMAP of cell type clustering from (**b**). (**d**) Dot plot of scaled normalized RNA expression for radial glial and neuronal markers across cell classes. (**e**) Frequency of cell classes (*left*) and total numbers of cells sequenced (*right*) (*n* = 3 independent differentiations, mean ± SEM). (**f**) Feature maps of normalized RNA expression plotted as UMAP for radial glial and neuronal markers.

iPSCs were stimulated with a combination of small molecules and recombinant proteins (chemical induction), cultured for an extended period of 10 weeks for maturation, and submitted for 10x sequencing (Fig. 1a). Molecularly distinct cell types were aligned by integrated cell clustering across 3 independent differentiation batches, and we classified 3 broad cell classes. Cell classes were defined by a discriminatory cell surface marker code: cluster of differentiation 99^+^ (CD99^+^), CD9^+^/CD24^+^, and neural cell adhesion molecule 1^+^ (NCAM1^+^) (Fig. 1b–d). Cell type proportions were determined across differentiations: 72.6% ± 5.2% CD99^+^, 6.4% ± 3.0% CD9^+^/CD24^+^, and 21.0% ± 6.3% NCAM1^+^ (mean ± SEM, *n* = 3 independent differentiations). Integrated analysis of cell type clusters resulted in total cell numbers of 16,366 CD99^+^, 5,207 NCAM1^+^, and 1,293 CD9^+^/CD24^+^ (Fig. 1e). We also validated the presence of these populations by staining cell class surface markers and observed similar trends in the percentages of differentiated populations (Supplementary Fig. 1). CD99^+^ cells expressed known progenitor restricted radial glial markers including SRY-box transcription factor 2 (SOX2), solute carrier family 1 member 3 (SLC1A3), nestin (NES), fatty acid binding protein 7 (FABP7), and vimentin (VIM). NCAM1^+^ cell types were depleted in these progenitor markers and enriched in neuronal markers including microtubule associated protein tau (MAPT) and doublecortin (DCX). CD9^+^/CD24^+^ cell class weakly expressed both neuronal and progenitor markers (Fig. 1d, f). To further characterize the NCAM1 cell class, we re-clustered this population according to molecularly distinct neurotransmitter codes (Supplementary Fig. 2). We found a neurotransmitter diversity of neurons expressing either SLC17A6 (VGLUT2) or SLC32A1 (VGAT) with a bias towards glutamatergic composition (Supplementary Fig. 2a–c). Additionally, we detected a VGLUT2^+^ population that co-expressed some dopaminergic neuron genes: dopa decarboxylase (DDC), nuclear receptor subfamily 4 group A member 2 (NR4A2), and LIM domain only 3 (LMO3) (Supplementary Fig. 2c). Our data indicates reproducible cell type outcomes resulting from chemical induction of iPSCs, and these types are molecularly heterogenous in the expression of neuronal and radial glial progenitor genes.

**Figure 2:**
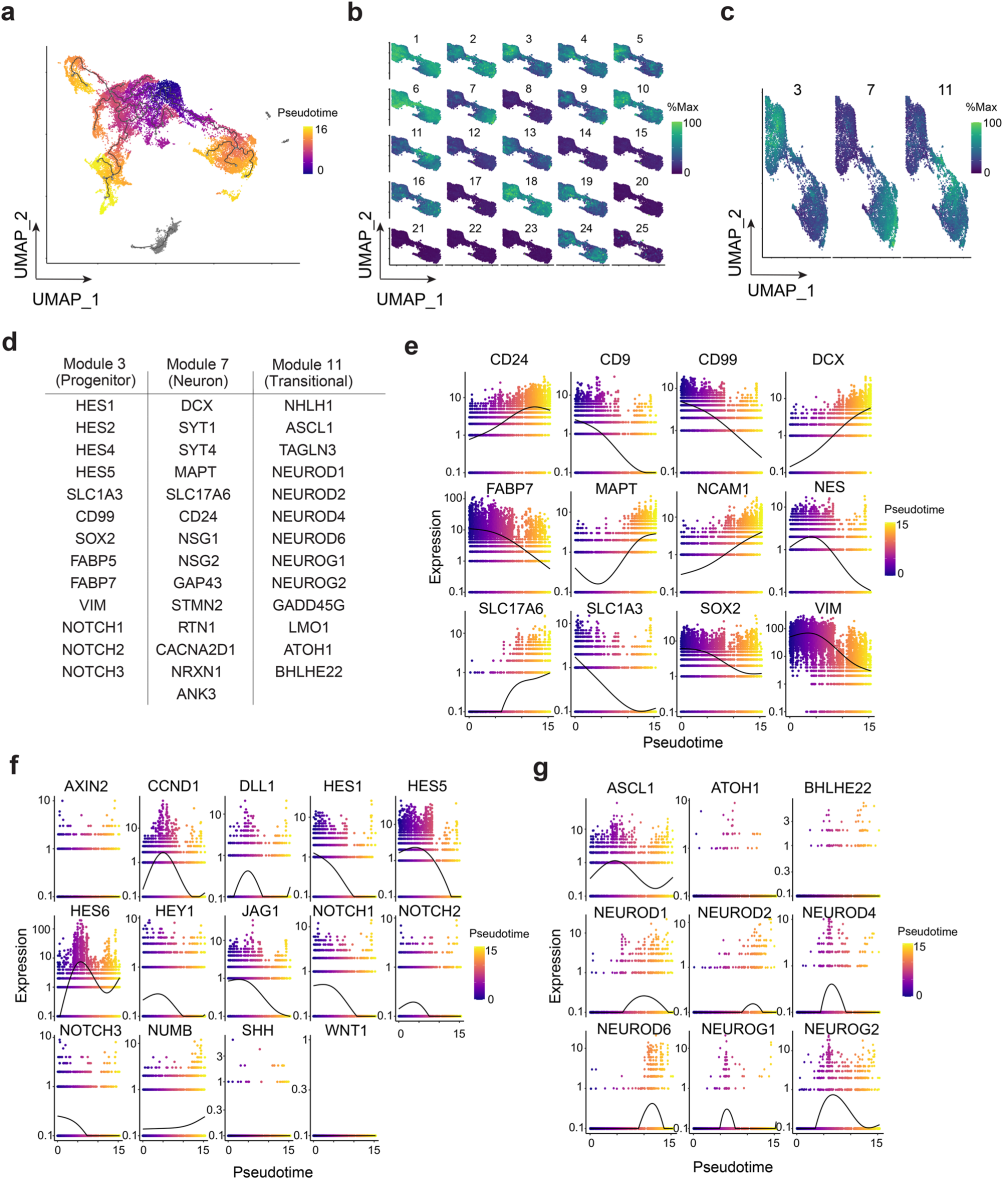
Single cell trajectory analysis of a human iPSC-derived neural mixed culture. (**a**) UMAP of learned trajectory graph from integrated cell type clustering with cells ordered in pseudotime. (**b**) Co-regulated gene modules of predicted lineage subset in (a) and visualized as scaled expression to percent of maximum expression. (**c**) Gene modules from (b) defined as progenitor (module 3), neuronal (module 7), and transitional state (module 11). (**d**) Table of example genes expressed in modules from (c). (**e**) Expression of radial glial and neuronal markers varying as a function of pseudotime on lineage subset from (b). (**f**) Expression of Notch, Shh, and Wnt related pathway genes as a function of pseudotime on lineage subset from (**b**). (**g**) Expression of gene module 11 transitional state bHLH transcription factors as a function of pseudotime on lineage subset from (**b**).

This heterogeneity of immature and mature neural markers prompted us to ask whether the asynchronous nature of chemically induced iPSC differentiations could inform intrinsic regulators of neurogenesis. We therefore modeled this mixed culture system as single cell differentiation trajectories with Monocle3. Using progenitor restricted radial glial markers as a starting cellular state, we constructed putative cellular lineages by organizing cells’ transcriptional states along pseudotime (Fig. 2a). We identified one lineage as a radial glial-neuron conversion by analyzing co-regulated gene modules that vary over pseudotime (Fig. 2b). Modules were analyzed using Monocle3 by performing UMAP on genes and then Louvain community analysis to organize genes into groups of “co-regulated gene modules.” We focused our analysis on modules 3, 7, and 11 because module 3 was concentrated at the beginning of pseudotime, module 11 was intermediate pseudotime, and module 7 was towards the end of pseudotime (Fig. 2a–c). Specific co-regulated gene modules were enriched in progenitor genes (module 3), neuronal genes (module 7), and neurogenic transcription factors (module 11) (Fig. 2c, d). We defined cells enriched in module 11 genes as transitional state cells that were transitioning out of a radial glial state into a neuron state. Analysis of neural markers within this lineage showed that as pseudotime increases, expression of neuronal markers increased with a complementary decrease in radial glial markers suggesting our lineage model recapitulates some aspects of neurogenesis (Fig. 2e).

We further explored the molecular underpinnings of this neurogenic lineage by performing a pathway analysis on Notch related genes and gene ontology mapping of module 11 transitional state cells. Consistent with Notch regulated neurogenesis, there was a decrease in expression of Notch transcription factors, such as hes family bHLH transcription factor 1 (HES1) and hes family bHLH transcription factor 5 (HES5), and Notch receptors, including NOTCH1 and NOTCH2, with increasing pseudotime. Genes related to other signaling pathways such as SHH and WNT1 were not reliably detected (Fig. 2f).

While our differentiation trajectory analysis suggests Notch signaling as a key upstream pathway that is downregulated during neurogenesis, we also analyzed other genes that were complementary induced with Notch downregulation over pseudotime. We hypothesized that module genes expressed in the transitional state cells are activators of neurogenesis. Analysis of module 11 transcription factors revealed several bHLH transcription factors and proneural genes. We observed ASCL1 and the NEUROD family of transcription factors were expressed in module 11. These transcription factors were more robust in expression compared to other bHLH transcription factors such as atonal bHLH transcription factor 1 (ATOH1) and basic helix-loop-helix family member e22 (BHLHE22) suggesting specificity of bHLH expression in our system (Fig. 2g). Module 11 genes were then analyzed with the Molecular Signatures Database (MSigDB) to compute overlaps with other gene sets. We found that our gene set overlapped with MSigDB gene sets including Neurogenesis, Neuron Differentiation, DNA Binding Transcription Factor Activity, and Neuron Development. ATOH1, ASCL1, NEUROD1, and NEUROD2 were the top bHLH transcription factors overlapping with these molecular signature gene sets (Supplementary Table 1). Collectively, our single cell trajectory analysis suggests our mixed culture model system recapitulates a Notch regulated neurogenesis. We identified putative proneural transcriptional regulators that might suppress Notch signaling and promote radial glial to neuron differentiation.

Directing neural precursor cells to select specific neurotransmitter identities has been attempted using various chemical induction approaches^1,5–12,35^. However, these protocols rely on activating/inhibiting promiscuous upstream developmental pathways that could result in heterogenous neurotransmitter gene expression. A better understanding of neurotransmitter regulation is needed to develop more efficient methods to direct neural progenitors into appropriate neurotransmitter identities. We therefore investigated the role of the transitional state transcription factors on regulating neurotransmitter selection with a focus on dopaminergic programming.

To prioritize our candidate genes from module 11 for gene-function experiments, we asked whether there were any correlations between these transitional state genes with dopaminergic neurotransmitter identity. We designed a strategy to profile dopaminergic pathway genes by building a genetic reporter for the expression of tyrosine hydroxylase (TH), the rate limiting enzyme in dopamine synthesis. Using CRISPR/Cas9 with homology directed repair (HDR), we inserted a P2A-tdTomato cassette into the 3’ endogenous locus of TH to purify live TH expressing cells with FACS (Supplementary Fig. 3a, b). Immunofluorescent analysis of purified cells demonstrated a polarized neural morphology and tdTomato^+^ cells were TH^+^ indicating reporter fidelity (Supplementary Fig. 3c, d).

**Figure 3:**
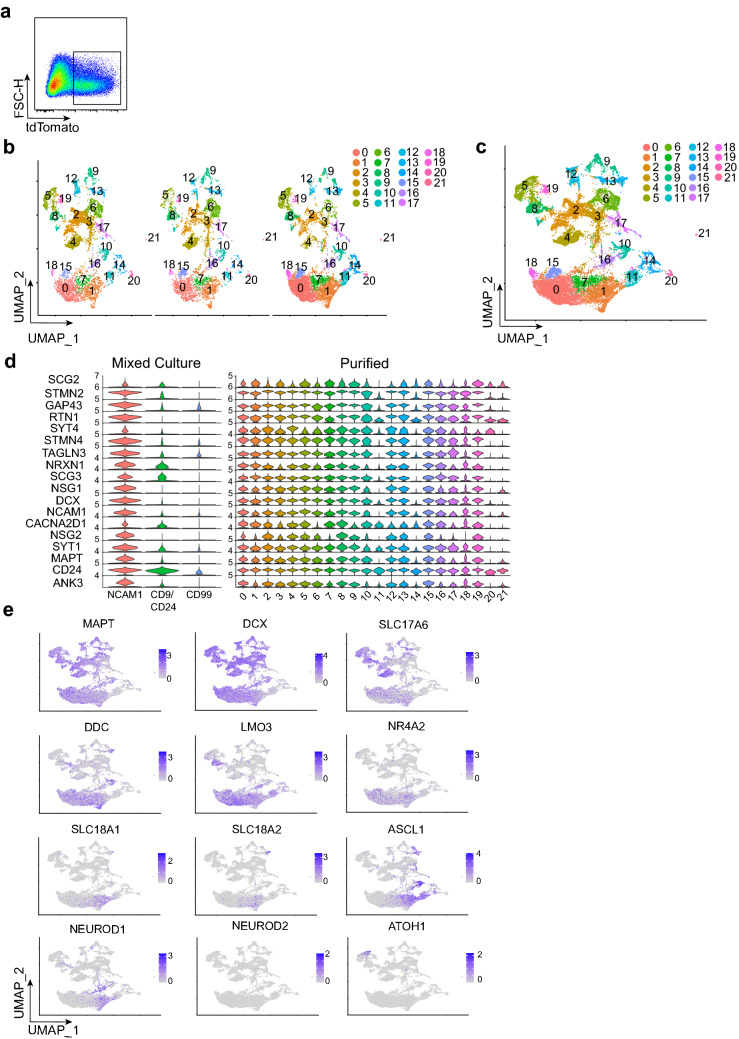
10x single cell RNA sequencing of FACS purified human TH expressing neurons programmed from iPSCs. (**a**) FACS plot of live TH expressing cells from a *Th-P2A-tdTomato* iPSC line. (**b**) UMAP of integrated cell type clustering from FACS purification with cell types numbered (*n* = 3 independent differentiations). (**c**) Merged UMAP of cell type clustering from (**b**). (**d**) Stacked violin plots of normalized RNA expression for neuronal markers comparing neural mixed culture cell classes to purified neuron cell type clusters. (**e**) Feature maps of normalized RNA expression plotted as UMAP for neuronal, glutamatergic, dopaminergic, and bHLH transcription factor genes.

TH expressing cells were then FACS purified at 5 weeks of culture during chemically induced differentiation for 10x single cell RNA sequencing to profile large numbers of neurons (purified neurons) (Fig. 3a). The percentage of TH expressing cells out of total differentiating cells was 29.3% ± 2.5% quantified by FACS (mean ± SEM, *n* = 3 independent differentiations) consistent with previous quantifications of this genetic background^36,37^ as well as the traditional floor plate derived dopaminergic protocol using a different genetic background^8^. Molecularly distinct cells were aligned by integrated cell clustering across these 3 independent differentiation batches (Fig. 3b, c). Comparison between our radial glial/neuron mixed culture system and purified neuron system revealed an enrichment of neuronal genes including DCX, MAPT, and synaptotagmin 1 (SYT1) (Fig. 3d). Many clusters of purified neurons expressed midbrain floor plate markers FOXA2 and LMX1A, which are known to be co-expressed in the traditional floor plate derived dopaminergic protocol^8^. These clusters also expressed pituitary homeobox 2 (PITX2) (Supplementary Fig. 4), normally expressed in the caudal diencephalon^13,38,39^. This is consistent with PITX2 upregulation in the traditional floor plate derived dopaminergic protocol compared to other neuron type differentiations^8^. TH purification resulted in high purity neurons and we confirmed at the single cell level, transcription factor expression of markers previously identified by other population based transcriptomic analyses^8^. These neurons were transcriptionally diverse, similar to another single cell RNA sequencing study^40^. However, further work is needed to correlate these purified neurons to in vivo counterparts.

We then determined correlational relationships between transcription factors and neurotransmitter gene expression in purified neurons. Of the top bHLH candidates identified from our mixed culture system, we detected ATOH1, NEUROD1, and ASCL1 expression. We observed some key genes that are expressed in human dopaminergic neurons: DDC, NR4A2, SLC18A1/2 (VMAT1/2), and LMO3. In the cell types co-expressing the most of these markers, NEUROD1 and ASCL1 were both co-expressed. ATOH1 was co-expressed with cells that lacked these particular dopaminergic traits but were positive for glutamatergic SLC17A6 (Fig. 3e). SLC17A6 was expressed in several cell type clusters and overlapped with dopaminergic neurotransmitter genes and transcription factors (Fig. 3c,e).  This co-expression has also been observed transiently in mouse dopaminergic neurons^41–43^. Analysis of additional module 11 transitional state transcription factors identified NHLH1 and NHLH2 as transcription factors expressed in SLC17A6^+^ cell types but excluded from the ASCL1/NEUROD1/ DDC/ NR4A2/ LMO3/ SLC18A1/2 expressing cell cluster (Supplementary Fig. 5). Collectively, the purified neuron 10x single cell RNA sequencing indicates specificity of transitional state transcription factor expression in distinct cell types. Additionally, this purified neuron data set shows that NEUROD1 and ASCL1 is biased to cell types expressing canonical floor plate markers and a more complete dopaminergic neurotransmitter identity.

**Figure 4:**
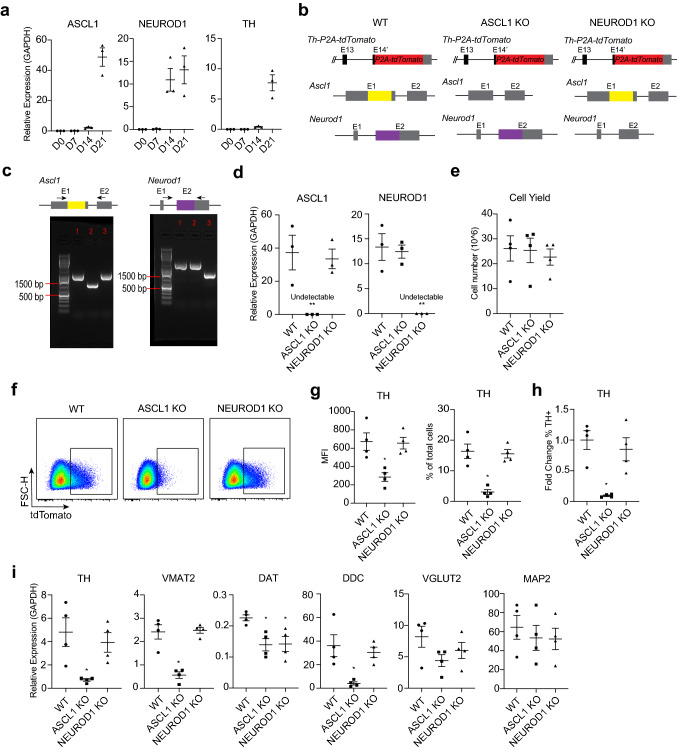
Distinct loss of function effects of ASCL1 and NEUROD1 on neurotransmitter selection in human iPSC-derived neurons. (**a**) RT-qPCR of culture temporal gene expression (*n* = 3 independent differentiations, relative gene expression normalized GAPDH). (**b**) Gene structure of iPSC KO lines; exons-boxes and introns-lines. ASCL1 coding sequence, yellow. NEUROD1 coding sequence, purple. (**c**) Gel electrophoresis of genomic DNA PCR for ASCL1 (*left*) and NEUROD1 (*right*); lane 1-WT, lane 2-ASCL1 KO, lane 3-NEUROD1 KO lines. (**d**) RT-qPCR of culture gene expression for ASCL1 and NEUROD1 for iPSC KO lines (*n* = 3 independent differentiations, relative gene expression normalized GAPDH, **Undetectable). (**e**) Trypan blue cell count of total cell numbers from dissociated cultures (*n* = 4 independent differentiations). (**f**) FACS plot of live TH expressing cells for WT, ASCL1 KO, and NEUROD1 KO lines. (**g**) Mean fluorescent intensity (*left*) and percentage TH^+^ expressing cell (*right*) quantification of FACS analysis from (**f**) (*n* = 4 independent differentiations). (**h**) Intracellular TH staining quantified by flow cytometry for WT, ASCL1 KO, and NEUROD1 KO lines (*n* = 4 independent differentiations). (**i**) RT-qPCR culture gene expression of dopaminergic, glutamatergic, and pan-neural genes for WT, ASCL1 KO, and NEUROD1 KO lines (*n* = 4 independent differentiations, relative gene expression normalized GAPDH). Error bars for (**a**, **d**, **e**, **g**, **h**, **i**), mean ± SEM. Kruskal Wallis test with Dunn’s post-hoc test for (**g**, **h**, **i**), *p < 0.05 significance compared to WT.

Because NEUROD1 and ASCL1 were co-expressed with many dopaminergic genes we hypothesized that these transcription factors regulate the iPSC-derived dopaminergic phenotype. We first assessed the temporal expression pattern of NEUROD1 and ASCL1 with TH. Induction of NEUROD1 and ASCL1 were temporally coupled with the induction of TH expression, although NEUROD1 spiked in expression before ASCL1 (Fig. 4a). To test the hypothesis that NEUROD1 and ASCL1 are necessary for regulating the iPSC-derived dopaminergic phenotype, we engineered global iPSC knockout (KO) lines by using CRISPR/Cas9 and non-homologous end joining (NHEJ) to separately delete the coding sequences of NEUROD1 and ASCL1 in our TH reporter system (Fig. 4b–d). ASCL1 KO and NEUROD1 KO with control WT lines were then differentiated into TH neurons and analyzed by FACS. Quantification of differentiated cell yields from dissociated preparations at the time of TH induction showed that ASCL1 KO and NEUROD1 KO did not result in changes in cell numbers, thus arguing against significant cell death or excessive proliferation in these mutants (Fig. 4e). Using FACS, we analyzed the mean fluorescent intensity (MFI) of TH expressing neurons in differentiating cultures and found a decrease in ASCL1 KO compared to WT (58.0% ± 3.3% decrease, mean ± SEM paired *n* = 4 independent differentiations) but not NEUROD1 KO. Analysis of the percentage of TH^+^ neurons out of total differentiating cells was determined to be decreased in ASCL1 KO compared to WT (82.2% ± 2.1% decrease, mean ± SEM paired *n* = 4 independent differentiations) but not NEUROD1 KO (Fig. 4f, g). Furthermore, we stained for TH and observed selective defects in the induction of TH protein for ASCL1 KO but not in NEUROD1 KO (Fig. 4h, Supplementary Fig. 6).

We then evaluated the population level gene expression in differentiating cells in ASCL1 KO and NEUROD1 KO compared to WT during TH neuron programming. RNA expression of dopaminergic genes TH, VMAT2, DDC, and the dopamine transporter (DAT) were measured in these cultures by RT-qPCR. While we were unable to reliably detect DAT by 10x single cell RNA sequencing, we detected low baseline culture expression of DAT compared to other dopaminergic traits. There was a mild decrease in DAT expression for both ASCL1 KO (39.1% ± 6.3% decrease) and NEUROD1 KO (36.3 ± 12.4% decrease) (mean ± SEM paired *n* = 4 independent differentiations). However, we observed more prominent decreases in expression for TH (82.9% ± 3.1% decrease), VMAT2 (77.9% ± 3.8% decrease), and DDC (89.3% ± 1.4% decrease) in ASCL1 KO cultures (mean ± SEM paired *n* = 4 independent differentiations). Because our 10x single cell RNA sequencing showed an overlap of neurons expressing dopaminergic genes and glutamatergic genes, we asked whether there was any effect on glutamatergic identity in our ASCL1 KO or NEUROD1 KO lines. VGLUT2 expression was not significantly changed and neither was pan-neural MAP2, suggesting that ASCL1 and NEUROD1 are largely dispensable for glutamatergic identity and some aspects of neural induction (Fig. 4i). Collectively, our loss of function experiments demonstrate specificity of human bHLH function in regulating neurotransmitter selection and ASCL1 is important for regulating the iPSC-derived dopaminergic phenotype.

## Discussion

Here, we used single cell RNA sequencing to screen for transcription factor drivers in iPSC-derived neurogenesis to elucidate the human molecular logic of neurotransmitter acquisition. We established a chemically defined directed differentiation method that promotes high efficiency progenitor cells expressing radial glial markers and demonstrate the utility of this system as a model of human neurogenesis. Neurogenic lineage construction uncovered a gene module containing bHLH transcription factors as putative regulators of radial glial to neuron conversion. Using correlational analysis of single cell RNA sequencing dopaminergic genes with bHLH transcription factors as a guide for gene-function experiments, we provide evidence that ASCL1 functions in acquisition of the dopaminergic phenotype. We also demonstrate that the human iPSC-derived dopaminergic phenotype requires specific bHLH transcription factor expression.

We provide the first study to our knowledge of bHLH transcription factor loss of function in human iPSC-derived dopaminergic programming. bHLH transcription factors have been implicated in regulating neuron subtype specification and used in overexpression studies to direct neuron type fates^4,44–50^. It is known that both ASCL1 and NEUROD1 overexpression facilitates acquisition of neuronal programs^4,26^. These bHLH transcription factors with other transcription factor cocktail combinations can also induce some dopaminergic traits^51–53^. Our human knockout data showing selective dopaminergic deficits supports the view that bHLH transcription factors are not only acting as generic pan-neuronal inducers but regulate distinct molecular programs.

During mouse dopaminergic development, some studies have examined the role of bHLH transcription factors such as NEUROG2 (NGN2), ASCL1, and NEUROD1. In ASCL1 knockout mice, midbrain dopaminergic neuron differentiation is preserved^30^, whereas hypothalamic dopaminergic neurons are depleted^32^. Mouse midbrain dopaminergic neurons are rather more dependent on the bHLH determinant NGN2, since NGN2 KO causes severe defects in midbrain dopaminergic neurogenesis and differentiation^30,31^. These deficits are exacerbated in a double NGN2 KO and ASCL1 KO background^30^, suggesting that ASCL1 can partially compensate for the loss of NGN2. Consistent with this, when ASCL1 is expressed from the NGN2 endogenous locus, dopaminergic neurogenesis is partially rescued^30^. Together, this data suggests that ASCL1 and NGN2 have partially redundant roles in dopaminergic neuron development. For NEUROD1, it is unclear what role this transcription factor has in dopaminergic neurogenesis. However, in the double NEUROD1 KO and NEUROD6 KO mouse, a subset of ventral tegmental area (VTA) neurons show reduced survival^54^. Extending these ASCL1 and NEUROD1 knockout studies in mice, we present evidence for a human differentiation platform where the induction of dopaminergic neurotransmitter genes is ASCL1 dependent.

While the specific molecular mechanism of bHLH regulation of dopaminergic neurotransmitter selection was not addressed in this study, we propose some models that should be addressed in future studies. Our experiments do not exclude potential indirect effects of ASCL1 regulation during neurotransmitter selection. It is possible that ASCL1 knockout disrupts a progenitor pool, which then fails to differentiate into neurons expressing dopaminergic neurotransmitter genes. This altered progenitor pool may default into a glutamatergic lineage, which can explain the biased dopaminergic deficit in ASCL1 KO but a relatively preserved glutamatergic identity (Fig. 4i). Alternatively, ASCL1 may also function by directly regulating the chromatin landscape of the dopaminergic neurotransmitter modules, providing access for downstream transcription factors to engage their gene targets. ASCL1 may execute this function by regulating epigenetic modifications^49^.

In our study, we observed specificity of bHLH transcription factors in regulating neurotransmitter selection. Some evidence suggests that the helix-loop-helix domains of ASCL1 and NEUROD1 can regulate different effects on dopaminergic programs^55^. Recent comparison of ASCL1 and NEUROG2 bHLH transcription factors shows that DNA E-box motif variants can determine unique chromatin binding preferences and regulate subtype transcriptional networks^49^. ChIP-seq data on human cancer cell lines comparing ASCL1 and NEUROD1 transcriptional targets demonstrates that these two bHLH genes bind to distinct genomic regions^56^. ASCL1 preferentially bound regions include neurotransmitter genes such as DDC, and these sites share some molecular target conservation between human and mouse^56^. Thus, non-overlapping binding site specificity may at least in part explain the biased necessity of ASCL1 relative to NEUROD1 for regulating the dopaminergic program.

One limitation of our study is the lack of cellular lineage tracking tools. Combined single cell RNA sequencing with cellular barcoding approaches may provide an even deeper understanding into how specific bHLH loss of function in iPSC-derived neurons dysregulates neuron commitment or neurotransmitter selection. Additionally, lineage tracking tools may help validate our single cell differentiation trajectories. Our trajectories are predicted lineages based on known immature and mature molecular markers. Further studies are needed to elucidate progenitor to neuron lineages in this model of human neurogenesis. Despite this limitation, we provide a single cell resource of putative transcription factors that may be useful for boosting efficiency in directing chemically primed neural progenitors into neurons.

How gene regulatory networks influence decisions of uncommitted neural progenitors to specialize into diverse neuron types is a fundamental question in developmental neurobiology. Here, we further our understanding of gene regulatory networks involved in dopaminergic neuron specification. These findings provide important insights towards the goal of improving dopaminergic neuron programming from hPSCs and have implications for developing clinically relevant models for Parkinson’s disease modeling and transplantation therapies^57–62^. By using a human pluripotent stem cell model system, we demonstrate how single cell approaches and gene targeting technology can help elucidate the molecular regulation of human neuron development.

## Materials and methods

### Human iPSC and neural differentiation culture

Human dermal fibroblasts from a healthy subject were donated from the University of Lübeck, Germany^63^. Fibroblasts were reprogrammed into the iPSC line, 2131, and were previously characterized^36,37,64,65^. iPSCs were grown in feeder-independent mTeSR1 (STEMCELL Technologies) on hESC-qualified Matrigel matrix (Corning 354277) according to manufacture recommended culture protocol. iPSCs were routinely tested negative for mycoplasma contamination. For maintenance, iPSCs were passaged with PBS 0.5 mM EDTA as aggregates. For neural mixed culture differentiation, iPSCs were chemically induced with a modified dual-SMAD differentiation^1,5,8^. iPSCs were dissociated into single cells by Accutase (Sigma A6964) and plated in mTeSR1 and 10 μM Rock inhibitor (Y) (Y-27632, Tocris 1254) at a density of 74,000 cells/cm^2^ on hESC-qualified Matrigel matrix coated at 2x the manufacture recommended coating protocol to promote long term high density cellular attachment (coated 4 °C overnight). Y was removed 24 h after plating and iPSCs were expanded in mTeSR1 until reaching high density confluency. On the start day of differentiation, differentiation day 0 (D0), media was changed to a differentiation medium composed of KnockOut DMEM (Invitrogen 10829018) supplemented with 15% Knockout Serum Replacement (KOSR) (Invitrogen 10828028), 1x MEM NEAA (Invitrogen 11140050), 1x Glutamax (Invitrogen 35050061), 2-Mercaptoethanol 100 μM (Invitrogen 21985023), and 50 U/mL Penicillin–Streptomycin (P/S) (Invitrogen 15140122). D0-D13 media was changed daily. Differentiation medium was supplemented with dual-SMAD inhibitors 100 nM LDN-193189 (LDN) (Reprocell 04-0074) and 10 μM SB-431542 (SB) (Tocris 1614) on D0. On D1-D2 differentiation medium contained LDN + SB with neural patterning factors 100 ng/mL Shh-C24II (R&D Systems 1845-SH-100), 2 μM Purmorphamine (Purm) (Reprocell 04-0009), 100 ng/mL FGF8a (R&D Systems 4745-F8-050). On D3-D4 differentiation medium contained LDN + SB + Shh-C24II + Purm + FGF8a and 3 μM CHIR-99021 (CHIR) (Reprocell 04-0004). On D5-D6, differentiation medium was reduced to 3:1 with a neural media composed of Neurobasal (Gibco 21103049), 1 × NeuroCult SMI Neuronal Supplement (STEMCELL technologies 05711), 1 × Glutamax, and 50 U/mL Penicillin–Streptomycin (3:1 adaption media). D5-D6 3:1 adaption medium contained LDN + Shh-C24II + Purm + FGF8a + CHIR. On D7-D8 differentiation medium was reduced to 1:1 with neural media (1:1 adaption medium) supplemented with LDN + CHIR. On D9-D10 differentiation medium was reduced to 1:3 with neural media (1:3 adaption medium) supplemented with LDN + CHIR. On D11-D12 neural media was supplemented with CHIR + 20 ng/mL BDNF (R&D Systems 248-BDB-050/CF), 20 ng/mL GDNF (R&D Systems 212-GD-050), 1 ng/mL TGFB3 (R&D Systems 8420-B3-005), 500 μM cAMP (Enzo Lifescience BML-CN125), 200 μM Ascorbic Acid (AA) (Sigma A5960), and 10 μM DAPT (Reprocell 04-0041). D13 differentiating cells were passaged 1:1 as large cell clusters *en bloc* to pre-coated dishes of 4 μg/cm^2^ Poly-L-ornithine (Sigma P4957)/ 3 μg/cm^2^ Laminin (Invitrogen 23017015) in neural media + BDNF/GDNF/TGFB3/cAMP/AA/DAPT (neural media complete). Neural media complete was fully changed every other day until week 3 of differentiation at onset of neural polarization. Cells were then replated by Accutase dissociation into single cells at high density conditions of 315,000 cells/cm^2^ in neural media complete + Y. The following day neural media was fully changed and Y was removed. Neuralized cells were then cultured in neural media complete with half media changes every 2–3 days until D40 when BDNF/GDNF/TGFB3/cAMP/AA/DAPT were removed. Neural media was used for half media changes every 2–3 days until harvested for 10x single cell sequencing at 10 weeks of culture.

For dopaminergic neuron differentiation, iPSCs were grown with an adapted TH neuron induction protocol^8^ on feeder-dependent MEF culture in a stem cell media composed of DMEM/F-12 (Invitrogen 11330057) supplemented with 20% KOSR, 1x MEM NEAA, 1x Glutamax, 50 U/ml P/S, 100 μM 2-Mercaptoethanol, and 4 ng/mL bFGF (Gibco PHG0266). CF1 IRR MEFs (Gibco A34181) were plated in DMEM (Invitrogen 11995073) + 10% FBS at 18,000 cells/cm^2^ on 0.2% Gelatin (Sigma 1890) coated plates for iPSC culture and 36,000 cells/cm^2^ for conditioned MEF media. Conditioned MEF media was generated by culturing MEFs with stem cell media with daily media collections for 5 days. For iPSC feeder dependent maintenance, iPSCs were passaged with Dispase (STEMCELL Technologies 07923) as aggregates. Before differentiation when iPSC colonies were large and compact, MEFs were aspirated first by manual removal. MEFs were further depleted by panning on 0.2% gelatin coated plates for 45 min 37 °C following Accutase single cell dissociation of iPSC-MEF culture in stem cell media + Y. Enriched iPSCs were plated at 36,000 cells/cm^2^ in conditioned MEF media + 10 ng/mL bFGF + Y onto 40 μg/cm^2^ Matrigel (Corning 354234) coated plates (coated 4 °C overnight). Day after plating, Y was removed and iPSCs were expanded in conditioned MEF media + 10 ng/mL bFGF until high density confluency. On D0 media was changed to differentiation media + 100 nM LDN and 10 μM SB. On D1 media was changed to differentiation media + 100 nM LDN/ 10 μM SB/ 2 μM Purm/ 100 ng/mL Shh-C25II (R&D Systems 464-SH). On D3 media was changed to differentiation media + 100 nM LDN/ 10 μM SB/ 2 μM Purm/ 100 ng/mL Shh-C25II/ 3 μM CHIR. On D5, differentiation medium was reduced to 3:1 adaption with an N2 medium composed of DMEM/F-12 + 20 nM progesterone (Sigma P8783), 100 μM putrescine (Sigma P5780), 30 nM sodium selenite (Sigma S5261), 25 μg/mL insulin (Sigma I9278), 100 μg/mL apotransferrin (Sigma T1147), and 50 U/mL P/S. D5 3:1 adaption media was supplemented with 100 nM LDN/2 μM Purm/ 100 ng/mL Shh-C25II/ 3 μM CHIR. On D7, differentiation media was reduced to 1:1 with N2 media (1:1 adaption media) supplemented with 100 nM LDN and 3 μM CHIR. On D9, differentiation media was reduced to 1:3 with N2 media (1:3 adaption media) supplemented with 100 nM LDN and 3 μM CHIR. On D11, media was changed to a media composed of Neurobasal, 1x B27 (Gibco 17504044), 1x Glutamax, 50 U/mL P/S supplemented with 20 ng/mL BDNF/ 20 ng/mL GDNF/ 1 ng/mL TGFB3/ 500 μM cAMP/ 200 μM AA/ 10 μM DAPT (B27 complete media) + 3 μM CHIR. On D13, differentiating cells were passaged 1:1 as large cell clusters *en bloc* to pre-coated dishes of 4 μg/cm^2^ Poly-L-ornithine/ 3 μg/cm^2^ Laminin in B27 complete media. D14-D18 full media changes every other day with B27 complete media. On D20 for iPSC knockout line phenotyping, cells were dissociated with Accutase and harvested for FACS, cell counts, and gene expression analysis. For single cell profiling, neuralized cells beginning D20 were cultured with half media changes every 2–3 days with B27 complete media until harvested at 5 weeks of culture for FACS purification 10x single cell RNA sequencing.

### FACS 10x single cell RNA sequencing

For neural mixed culture dissociation, cells were dissociated with TrypLE (Gibco 12605010) for 5 min 37 °C. For dopaminergic neuron culture, cells were dissociated with 0.25% Trypsin–EDTA (Gibco 25200056) for 5 min 37 °C. Dissociated cells were washed in PBS + 10% FBS and stained with live/dead DAPI (0.5 μg/ml) for 10 min on ice. Cells were strained with a 40μ filter in flow buffer of PBS + 2% FBS and live cells (neural mixed culture) and live TH^+^ cells (dopaminergic culture) were sorted with FACS Aria for library preparation using Single Cell 3’ Reagent Kit v3 (10 × Genomics, Chromium). Each differentiation batch was used for independent libraries and were sequenced on HiSeq4000. Single cell sequencing was analyzed using Seurat v3 and Monocle3^66^ in R following their recommended workflow. Cells were pre-processed by analyzing single high quality cells with < 10% mitochondrial genes and < 6000 features. A regularized negative binomial regression method was used for normalization^67^. Data was integrated using anchors cell pairwise correspondences^68^. Jackstraw method was used for determining principal components and a shared nearest neighbor graph constructed based on a Louvain-Jaccard algorithm. After optimizing modularity of the clusters, cell clusters were mapped on UMAP for visualization and gene expression analysis.

### Neural cell surface and intracellular immunostaining

Neural mixed culture and dopaminergic neuron cultures were differentiated as above and harvested at 3 weeks of culture for cell surface or intracellular staining. Neural mixed culture was dissociated with Accutase for 5 min 37 °C. Dissociated cells were washed in PBS + 2% FBS, strained with 40μ filter, and stained with a cell surface panel at 1 × 10^6^ cells/ 100 μL staining buffer for 30 min 4 °C. Staining buffer consisted of PBS + 2% FBS, 10 μM Y, 1:20 Alexa Fluor 488 anti-human CD56 (Biolegend 362518), 1:20 Brilliant Violet 421 anti-human CD99 (Biolegend 371312), 1:20 PE/Cyanine7 anti-human CD24 (Biolegend 311120), and 1:20 APC/Fire 750 anti-human CD9 (Biolegend 312114). Cells were then washed with PBS + 2% FBS and then analyzed with a BD LSRFortessa Flow Cytometer.

For dopaminergic neuron culture immunostaining, cells were dissociated with Accutase for 5 min 37 °C and processed as above for FACS. Dissociated cells were fixed in a Permeabilization/ Fixation buffer (0.1% Saponin PBS 4% Formaldehyde (Polysciences 04018)) for 30 min room temperature at 1 × 10^6^ cells/100 μL Permeabilization/ Fixation buffer. Fixed cells were washed in a Permeabilization/ Blocking buffer consisting of 0.1% Saponin PBS + 2% FBS. Intracellular stain was performed by incubating fixed cells with a primary antibody stain 4° C ON of 1:400 Rb-anti-TH (Calbiochem 657012) in Permeabilization/ Blocking buffer. Next day, cells were washed with Permeabilization/ Blocking buffer and then stained with secondary antibody 1:500 Donkey anti Rb 488 (Invitrogen A-21206) in Permeabilization/ Blocking buffer for 30 min room temperature. Cells were washed, resuspended in PBS + 2% FBS and analyzed with a BD LSRFortessa Flow Cytometer.

### RT-qPCR gene expression analysis

RNA from cell cultures was extracted using RNeasy Mini Kit (Qiagen 74104). High Capacity cDNA Reverse Transcription Kit (Applied Biosystems 4368814) was used for cDNA reaction. qPCR was performed using SsoAdvanced Universal SYBR Green Supermix (BioRad 1725271) on 7500 Fast Real-Time PCR System (Applied Biosystems). Oligonucleotide primer sequences listed in Supplementary Table 2. Relative gene expression normalized GAPDH, or Relative Gene Expression (GAPDH), was calculated from the ${2}^{-d\;Ct}$, where $d\;Ct=Target\;gene\;Ct-GAPDH\;Ct$.

### Immunofluorescence microscopy

Cultures were fixed in 0.1% Saponin PBS 4% Formaldehyde (Polysciences 04018) for 15 min room temperature. Cells were washed with PBS and blocked with 0.1% Saponin PBS + 2%FBS for 30 min. Primary antibody stain was performed 4°C ON (1:400 TH Rb Calbiochem 657012; 1:1000 BIII tubulin Ms Biolegend 801201). Next day, cells were washed with 0.1% Saponin PBS + 2%FBS and stained with secondary antibodies (1:500 Donkey anti Rb 488 Invitrogen A-21206; 1:500 Donkey anti Ms 647 Invitrogen A-31571) for 30 min room temperature. Cells were washed, mounted with Prolong Diamond Antifade Mountant (Invitrogen), and imaged with Leica DMI4000B confocal microscope with Leica Application Suite X.

### CRISPR/Cas9 gene editing

For TH reporter engineering, TH-guide1 and TH-guide2 were cloned into PX461 (Addgene 48140). 2131 iPSC line was plated at 70,000 cells/cm^2^ in mTeSR1 + Y on hESC-qualified Matrigel. Next day, mTeSR1 was changed without Y and transfected with Lipofectamine 3000 (Invitrogen L3000008) at low Lipofectamine 3000 reagent condition according to manufacturer protocol. Plasmids transfected were PX461-TH-guide1, PX461-TH-guide2, and a donor vector containing TH homology arms with a P2A-tdTomato-pgk-neo cassette (Thermo GeneArt Gene Synthesis) at equal ratios following manufacturer guidelines. Day after transfection, replaced media with mTeSR1. On second day after transfection, begin selection with mTeSR1 + 200 μg/mL G418 (Gibco 10131035). Continue daily media changes with mTeSR1 + 200 μg/mL G418 for 1 week and then switched to mTeSR1. Cells were then plated at serial dilutions in + Y for clone pick to generate TH-P2A-tdTomato line. Following clonal plating Y was removed after 24 h. Reporter TH expression was confirmed by dopaminergic differentiation, FACS purification and fixed for TH/BIII immunofluorescence.

For ASCL1 and NEUROD1 iPSC knockout generation, 2 guides were used for cutting the beginning and end of the coding sequences for each gene: ASCL1-guide1, ASCL1-guide2, NEUROD1-guide1, NEUROD1-guide2. Guides were cloned into PX458 (Addgene 48138). TH-P2A-tdTomato line was transfected as described above with respective guides. After 48 h with daily mTeSR1 media changes, cells were dissociated with Accutase, FACS purified for GFP, and cultured in mTeSR1/1:200 anti-anti (Gibco 15240062)/Y. Enriched cells were plated at serial dilutions in + Y for clone pick to generate knockout lines. Following clonal plating Y was removed after 24 h. Oligonucleotide guide sequences listed in Supplementary Table 2.

## Supplementary Information

Supplementary Information 1.

Supplementary Information 2.

Supplementary Information 3.

Supplementary Information 4.

## Data Availability

The datasets generated during and/or analyzed during the current study are available from the corresponding author on reasonable request. GEO accession number for 10x single cell RNA sequencing: GSE185275. “Neural co-culture” in GEO metadata refers to neural mixed cultures.
