# Supplementary material for: Identification of ASCL1 as a determinant for human iPSC-derived dopaminergic neurons
Source: Sci Rep. 2021 Nov 15;11:22257. doi: 10.1038/s41598-021-01366-4 (PMC8593045; doi:10.1038/s41598-021-01366-4)
Supplement: Supplementary file 1 — Supplementary Information 1. [file 41598_2021_1366_MOESM1_ESM.pdf]

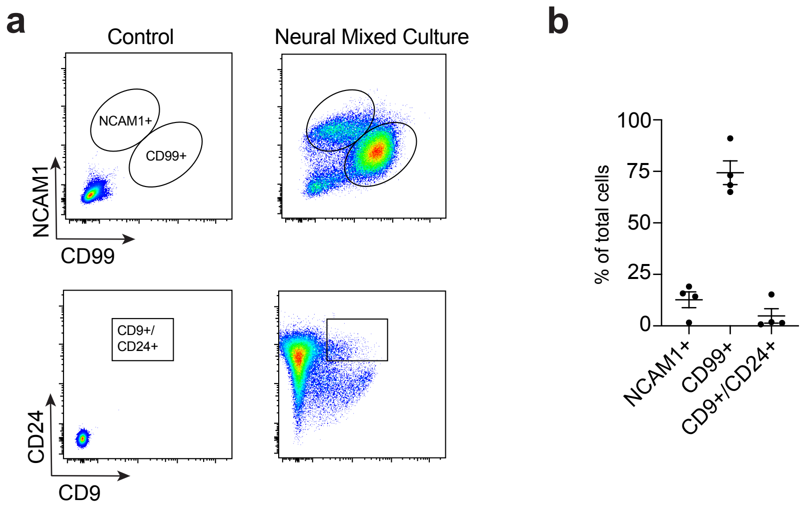

Supplementary Figure 1: Cell surface marker stain for neural mixed culture cell classes.
(a) Flow cytometry plots of neural mixed culture cell surface marker stain with unstained negative control.
(b) Quantification of population percentages in (a) for neural mixed culture (n = 4 independent differentiations, mean ± SEM).

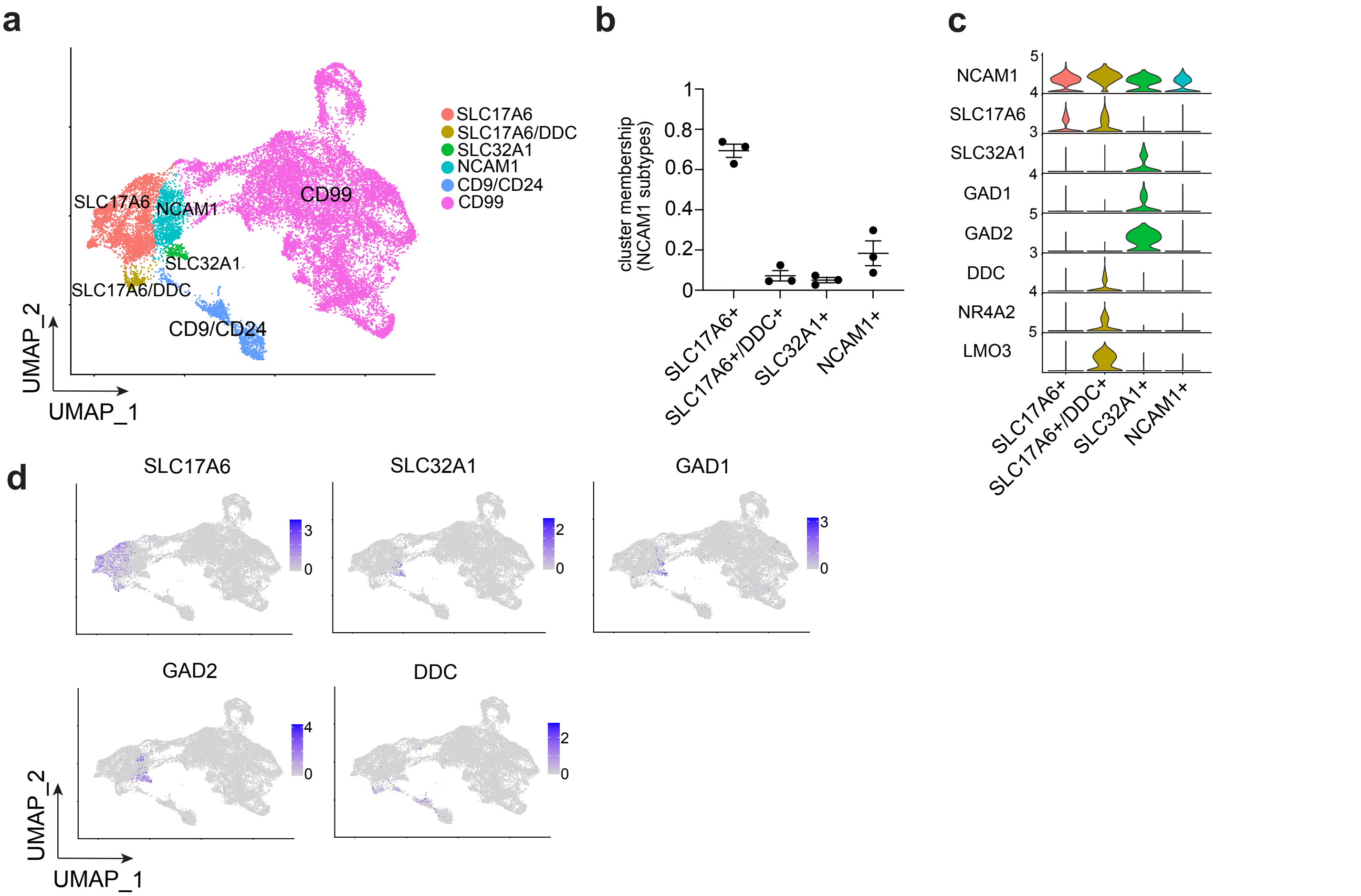

Supplementary Figure 2: Subtypes of neurotransmitter identities programmed in neural mixed culture differentiation.
(a) UMAP of cell type clustering corresponding to (Figure 1c) with neuronal subtypes of NCAM1 cell class (NCAM1 only subpopulation in this graph represents an unclassified neurotransmitter identity). **(b)** Frequency of neuronal subtype clusters from (a) out of total NCAM1 cell class (n = 3 independent differentiations, mean ± SEM). **(c)** Stacked violin plots of normalized RNA expression (gene by neuron subtype). **(d)** Feature maps of normalized RNA expression plotted as UMAP for neurotransmitter genes.

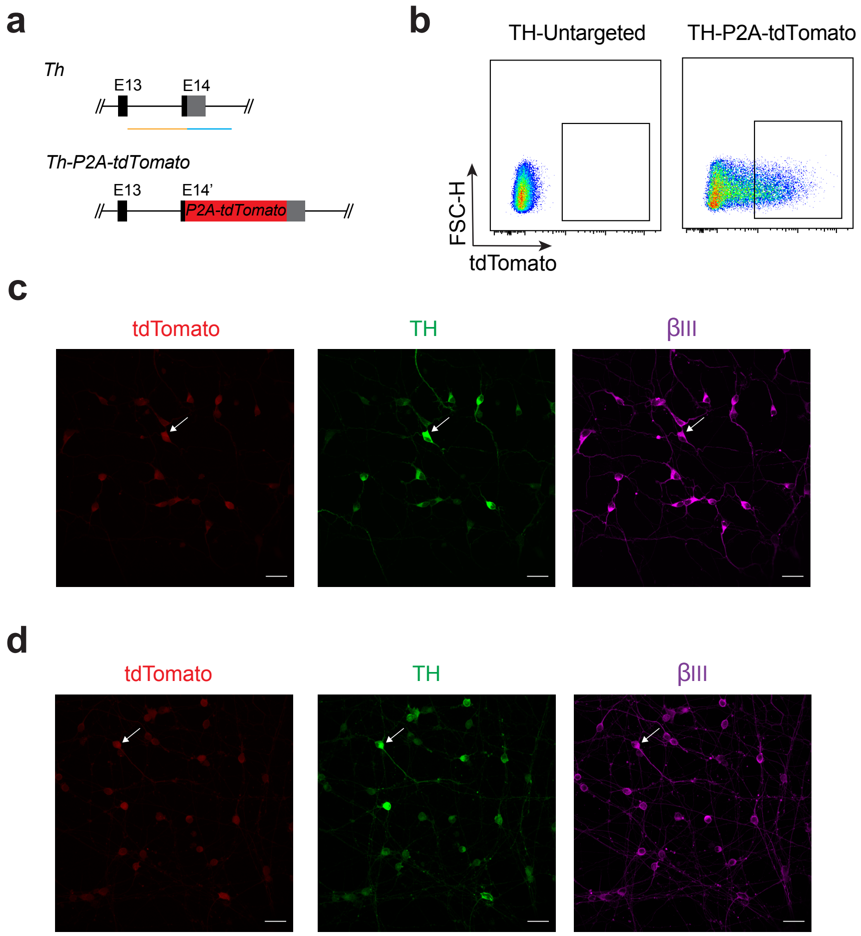

Supplementary Figure 3: Generation of a human iPSC TH-P2A-tdTomato line.
(a) Gene structure of untargeted TH (*top*) and targeted TH-P2A-tdTomato (*bottom*). Exons-boxes and introns-lines. **(b)** FACS validation of TH-P2A-tdTomato (*right*) with control untargeted line (*left*). **(c)** Immunofluorescence of 3 week FACS purified TH expressing neurons fixed 4 days post-sorting (3 weeks differentiated). Arrow indicates a tdTomato^{+} cell is TH^{+} (scale bar = 25µm). **(d)** Immunofluorescence of 3 week FACS purified TH expressing neurons fixed 2 weeks post-sorting (5 weeks differentiated). Arrow indicates a tdTomato^{+} cell is TH^{+} (scale bar = 25µm).

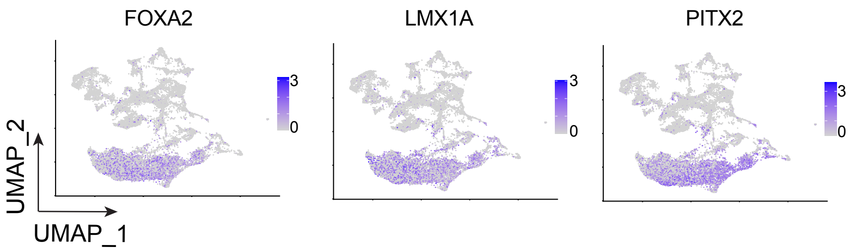

Supplementary Figure 4: Single cell expression of meso-diencephalic markers.
Feature maps of normalized RNA expression plotted as UMAP in purified neurons.

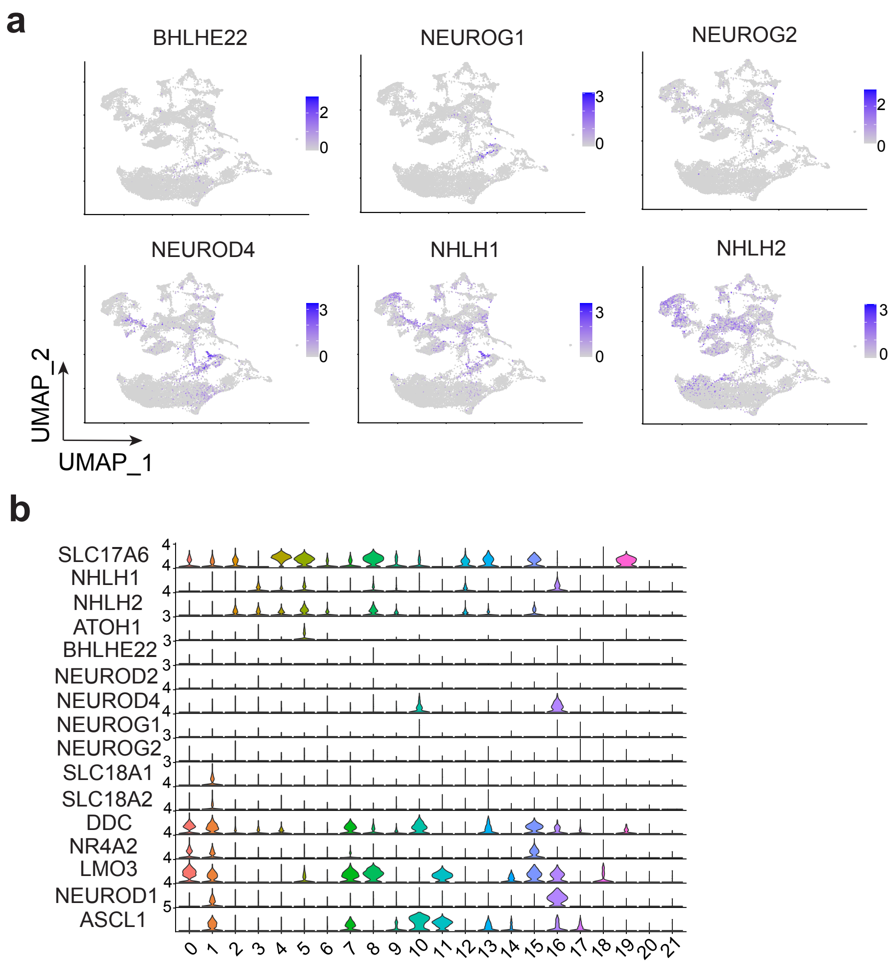

Supplementary Figure 5: Single cell expression of unprioritized bHLH transcription factors and relationship to prioritized genes.
(a) Feature maps of normalized RNA expression plotted as UMAP for unprioritized bHLH transcription factor candidates identified from module 11 transitional state genes. **(b)** Stacked violin plots of normalized RNA expression for module 11 transitional state candidate genes and cell cluster relationships with glutamatergic and dopaminergic genes (cluster numbers correspond to Fig. 3c).

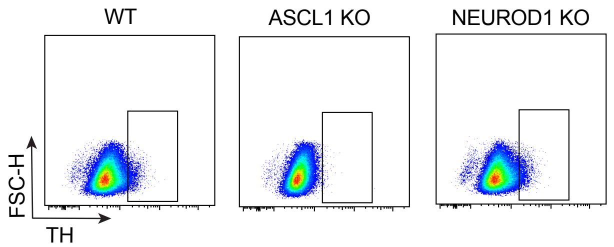

Supplementary Figure 6: Deficient TH induction in ASCL1 KO but not NEUROD1 KO.
Flow cytometry plots of intracellular TH stain.
