# Supplementary material for: Identification of ASCL1 as a determinant for human iPSC-derived dopaminergic neurons
Source: Sci Rep. 2021 Nov 15;11:22257. doi: 10.1038/s41598-021-01366-4 (PMC8593045; doi:10.1038/s41598-021-01366-4)
Supplement: Supplementary file 2 — Supplementary Information 2. [file 41598_2021_1366_MOESM2_ESM.docx]

**Supplementary Table S1: Gene Ontology Mapping.**

MSigDB gene set enrichment of gene module 11 transitional state cells.

**Supplementary Table S2: Oligonucleotide sequences.**

Oligonucleotide sequences for RT-qPCR, CRISPR-Cas9 guides, and genotyping. Sequences listed as 5’ 🡪 3’.
